# Prostate Cancer Survival by Risk and Other Prognostic Factors in Mallorca, Spain

**DOI:** 10.3390/ijerph182111156

**Published:** 2021-10-24

**Authors:** Juan José Montaño, Antoni Barceló, Paula Franch, Jaume Galceran, Alberto Ameijide, Jaime Pons, Maria Ramos

**Affiliations:** 1 Department of Psychology, University of Balearic Islands (UIB), 07122 Palma, Spain; 2 Health Research Institute of the Balearic Islands (IdISBa), 07120 Palma, Spain; pfranch@dgsanita.caib.es (P.F.); mramos@dgsanita.caib.es (M.R.); 3 Mallorca Cancer Registry, Balearic Islands Public Health Department, 07010 Palma, Spain; barcelo68@gmail.com (A.B.); jaume.pons@juaneda.es (J.P.); 4 Tarragona Cancer Registry, Cancer Epidemiology and Prevention Service, Hospital Universitari Sant Joan de Reus, Institut d’Investigació Sanitària Pere Virgili (IISPV), 43204 Reus, Spain; jaume.galceran@salutsantjoan.cat (J.G.); alberto.ameijide@salutsantjoan.cat (A.A.)

**Keywords:** prostate neoplasm, survival, stage, multiple imputation

## Abstract

Studies about the survival of patients with prostate cancer by stage or risk of progression are scarce. The aims of this study were (1) to determine the cause-specific survival by risk in prostate cancer patients in Mallorca diagnosed in the period 2006–2011; (2) to identify the factors that explain and predict the likelihood of survival and the risk of dying from this type of cancer; and (3) to determine the distribution of prostate cancer by risk in the patients in Mallorca diagnosed in the period 2006–2011. Incident prostate cancer cases diagnosed between 2006 and 2011 were identified through the Mallorca Cancer Registry. We collected age; date and method of diagnosis; date of follow-up or death; T, N, M and stage according to the TNM 7th edition; Gleason score; prostate-specific antigen (PSA); histology according to the International Classification of Diseases for Oncology (ICD-O) 3rd edition, comorbidities and treatments. We calculated risk in four categories: low, medium, high and very high. The end point of follow-up was 31 December 2014. Multiple imputation (MI) was performed to estimate cases with unknown risk. We identified 2921 cases. Five years after diagnosis, survival after MI was 89% globally, and was 100% for low-risk cases, 96% for medium risk, 93% for high risk and 69% for very-high-risk cases. Cases with histology other than adenocarcinoma, with high (and especially very high) risk, as well as with systemic, mixed and observation/unspecified treatments had worse prognoses.

## 1. Introduction

Prostate cancer is the most frequent cancer in men in the European Union and the second most frequent worldwide [1]. The Spanish Network of Cancer Registries (REDECAN) estimated an age-standardized incidence rate of 67.7 for 2020 [2]. Estimated age-standardized mortality rates are much lower: 10.2 for Europe and 7.5 for Spain for 2020 [1].

The EUROCARE-5 study estimated, for the 2000–2007 period, a 5-year relative survival rate for prostate cancer of 83.4% in Europe and of 84.7% in Spain [3]. Striking rises in 5-year relative survival of prostate cancer have occurred in Spain [4] and in many other countries, as the CONCORD-2 study reported for the 1995–2009 period [5]. Although a large part of this increase in prostate cancer survival in the general population has been due to the increase in the number of diagnoses of tumors with a very good prognosis caused by the widespread use of the prostate-specific antigen (PSA) test, both studies concluded that the information about stage only is not enough to explain the differences in survival observed among countries. 

Stage is the main prognostic factor in most cancers. However, for prostate cancer, the International Union Against Cancer (UICC) proposed in its 7th edition [6] a new classification based on risk or prognostic groups, using not only the T and N components of the stage, but also the histopathological grade or Gleason score and the prostate-specific antigen (PSA) value. There are many classifications of risk for prostate cancer that have different numbers of categories: three categories (low, medium and high) [7]; four (low, medium, high and very high) [8]; five (very low, low, medium low, medium high and high) [9]; and even seven (considering five localized categories, one for clinical lymph node involvement and one for disseminated metastases) [10]. Since stage of prostate cancer cannot be determined because N and M are frequently unknown, these new classifications involving risk stratification help to overcome the problem of stage as a missing value for prostate cancer. Population-based studies about the survival of patients with prostate cancer by stage or risk are scarce. Survival in prostate cancer is also associated with age [3], comorbidity [11] and treatment [12].

The aims of this study were (1) to determine the cause-specific survival by risk in prostate cancer patients in Mallorca diagnosed in the period 2006–2011; (2) to identify the factors that explain and predict the likelihood of survival and the risk of dying from this type of cancer; and (3) to determine the distribution of prostate cancer by risk in the patients in Mallorca diagnosed in the period 2006–2011. 

## 2. Materials and Method

### 2.1. Patient Involvement

This was a population-based retrospective follow-up study of patients living in Mallorca diagnosed with invasive prostate cancer between 2006 and 2011. We included all invasive prostate cancers of any histology according to the ICD-O (International Classification of Diseases for Oncology) 3rd edition (code C61.9) [6], identified through the Mallorca Cancer Registry. Cases exclusively identified through the death certificate only (DCO) and cases without follow-up were excluded.

### 2.2. Variables

Some of this study’s variables were already collected by the Mallorca Cancer Registry, such as age, date of diagnosis, basis of diagnosis, date of follow-up or death; T, N, M and stage according to the TNM 7th edition; Gleason score (differentiating the value 7 as 4 + 3 or 3 + 4); PSA and histology (according to the ICD-O 3rd edition) [13]. Other variables were collected by reviewing the Hospital and Primary Health Care clinical records, that is, comorbidities and all treatments received by the patient (prostatectomy, radiotherapy, cryosurgery, hormonal therapy, chemotherapy, active surveillance, expectant attitude and unknown).

Regarding stage, pathological T or N status was prioritized over clinical status in Mallorca Cancer Registries using an integrated approach [6] by combining pathological and clinical components, resulting in T_PT (pathological or clinical T) and N_PN (pathological or clinical N). When stage or risk was missing, the clinical records were also reviewed. 

Age was categorized in the following groups: 15–54, 55–64, 65–74, 75–84 and 85 years old and over. Life expectancy greater than or less than 10 years was also calculated according to the adjusted life expectancy at birth in the Balearic Islands [14]. Each individual survival time was defined from data of diagnosis to date of last known vital status (death by any cause, date of loss to follow-up, or date of end of follow-up on 31st December 2014). Vital status was categorized as: alive (0), dead by prostate cancer (1) or dead by other causes (2). 

We grouped PSA as: <10 ng/mL, 11–20 ng/mL, and >20 ng/mL. We calculated risk using T, N, Gleason and PSA, according to the criteria proposed by the European Association of Urology [8], and established four groups: low risk (T1-T2a and Gleason < 7 and PSA < 10), medium risk (T2b or Gleason = 7 or PSA between 10 and 20), high risk (T2c or Gleason > 7 or PSA > 20) and very high risk (T3-T4 or any T if N = 1). 

Histology was categorized as: adenocarcinoma (8140), including acinar (8550); without histology (8000); and others (8001, 8010, 8020, 8041, 8045, 8246, 8500). Non-epithelial tumors were not included in the study.

We calculated the Charlson Comorbidity Index and grouped the cases in four categories: 0, 1, 2 and ≥3. 

Treatments were grouped as follows: local (prostatectomy, radiotherapy and cryosurgery); systemic (hormonal therapy or chemotherapy); mixed (combination of local and systemic); and observation/unknown (active surveillance, expectant attitude and unknown). 

### 2.3. Statistical Analysis

Multiple imputation (MI) was used to obtain risk status when this was unknown, following three main steps [15]. First, we ran the imputation model and replaced each missing value with sets of 5, 10, 15 and 20 imputations by applying the multiple imputation chained equation (MICE) procedure. We assumed that the data were missing at random types. A more detailed description can be found in a previous manuscript [16]. Secondly, we analyzed the resulting imputed and complete data sets independently by applying a competing-risks regression. Finally, we applied a single competing-risks regression model using Rubin’s rules [17] from each set of 5, 10, 15 and 20 estimates resulting from the previous competing-risks regression model. We selected the MI with 10 imputations because the increase to 15 or 20 did not change the coefficient values, the standard errors or the degrees of significance. 

Prior to performing the survival analysis, the relationships between the variables were explored using contingency tables on which the Chi-square independence test [18] and the Cramer’s V association index [19] were calculated.

As we knew the cause of death, we used cancer-specific survival—that is, survival considering only prostate cancer as cause of death. However, we also calculated the relative survival by the Ederer II method [20], which considers other causes of death, using lifetables obtained from published official mortality data of the Balearic Islands [14]. Since the main population-based survival studies such as EUROCARE and CONCORD have used relative survival, we decided to calculate both types of survival to be able to compare them with each other and with the aforementioned studies.

We applied the survival analysis by the actuarial and Kaplan–Meier methods to estimate the likelihood of survival and risk of death; the log-rank test to evaluate the statistical differences of the observed survival curves by each categorical variable; their respective graphic representations to compare and observe the evolution of survival over time and the competing-risks regression models to identify the prognostic factors associated with mortality risk. 

Competing-risks regression [21] provides a useful alternative to Cox regression [22] for survival data in the presence of competing risks. Competing-risks regression posits a model for the subhazard function of a failure event of primary interest, in the presence of competing failure events that impede the event of interest. This is not to be confused with the usual right-censoring found in survival data, such as censoring due to loss to follow-up. While censoring merely obstructs from observing the event of interest, a competing event prevents the event of interest from occurring altogether. In our study, the event of interest was death from prostate cancer, while the competing failure event was death from other causes. Finally, this type of model estimates the subhazard ratios with an interpretation similar to the hazard ratios in the Cox regression.

Age, histology, risk, Charlson index and treatment were included in the competing-risks model, while stage, PSA and Gleason were excluded because they are part of the variable “risk”. Cases with low risk were also excluded because their survival rate was 100%. The proportional hazard assumption for each covariate was tested by introducing time-dependent variables. 

The procedure for selecting the covariates in the final competing-risks model was based on the Wald test. To compare the effect of the imputation procedure on the subhazard ratio estimation of covariates, the competing-risks regression model was performed both before and after MI.

We carried out MI with STATA 16, and survival analysis with Statistical Package for the Social Sciences (SPSS) 25.

## 3. Results

In total, 2921 cases of invasive prostate cancer were identified between 2006 and 2011. Stage was unknown in 53.9% of cases, while risk was unknown in 24.5%. After MI, the distribution of cases by risk was as follows: 9.5% had low risk, 24.9% medium risk, 42.7% high risk and 22.9% very high risk. A full description of the sample is shown in Table 1.

Survival analysis was performed with 2917 cases because vital status was unknown for 4 cases. At the end of the study, a total of 2244 (76.8%) patients had survived, 324 (11.1%) died of prostate cancer, and 349 (12%) died from other causes. Average time of survival was 2954 days (CI 95% = [2920, 2987]), with a standard error of 17.19.

Table 2 presents the relationship of the treatment variable with the variables whose relationship was more relevant. Treatment showed a more relevant relationship with age and life expectancy than with risk and Charlson index and histology (adenocarcinoma, unspecified and others).

Table 3 shows no changes in cancer-specific survival rates by year after MI for the whole sample. However, survival percentages by risk were slightly higher after MI, especially in very-high-risk cases; thus, without MI, survival by risk and year would have been slightly underestimated. At 5 years after diagnosis, survival after MI was 89% for the whole sample; it was 100% for low-risk cases, 96% for medium-risk cases, 93% for high-risk cases and 69% for very-high-risk cases.

Table 4 displays 5-year relative survival by risk before and after MI. As cancer-specific survival, relative survival did not show changes for total cases between before and after MI with a value of 91%. Slightly higher survival rates were also observed in very-high-risk cases after MI.

**Figure 1:**
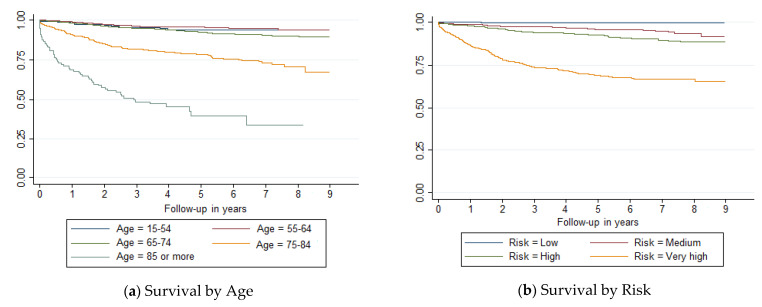

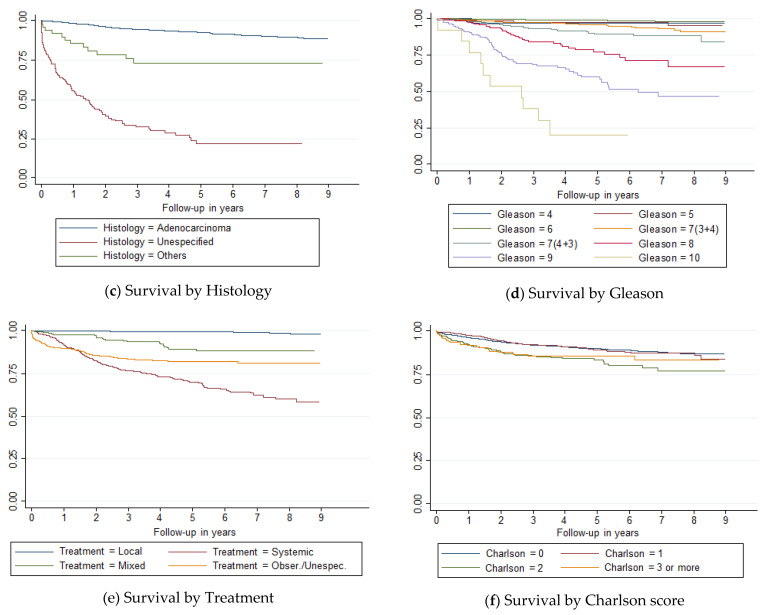
Survival curves of prostate cancer cases diagnosed in Mallorca between 2006 and 2011 by (**a**) age, (**b**) risk, (**c**) histology, (**d**) Gleason, (**e**) treatment and (**f**) Charlson score.

Survival curves showed differences (*p* < 0.001) by age, risk, histology, Gleason score and treatment (Figure 1). Prostate cancer survival diminished markedly in people over 75 years old, with very high risk, histology other than adenocarcinoma, PSA values at diagnosis >20, Gleason values ≥8, or after a systemic treatment. However, there was no clear relationship between Charlson index and survival.

The Wald test included Charlson index, risk, histology and treatment in the final competing-risks model. Age was excluded because of the detected relationship between age and treatment. Table 5 shows the results of the competing-risks model before (Model 1) and after (Model 2) MI. Both models (original vs. MI) determined that patients with a Charlson index of 2 had worse prognosis than those with an index of 0; however, those with an index of 3 or more did not have a worse prognosis than those with an index of 0. Moreover, patients with histology other than adenocarcinoma; with high risk (especially very high risk); and with systemic, mixed and observation/unspecified treatments also had worse prognosis. In general, standard errors were lower after MI, providing more accurate estimates of the risk of dying from this cancer.

## 4. Discussion

The prostate cancer 5-year relative survival rate in Mallorca was 91% for the patients diagnosed in the period 2006–2011, which is close to the one found by the CONCORD-3 study for 10 Spanish registries for the period 2005–2009 (90.4%) [23], and slightly higher than the one found by the EUROCARE-5 study for Spain for the previous period 2000–2007 (84.6%) [3].

On the other hand, the 5-year cancer-specific survival rate (89%) was two percentage points lower than the relative survival. Both measures of survival are widely used in medical research, but neither methodology achieves a perfect net survival estimate and therefore the strengths and limitations of both methodologies must be known. The main limitation of cancer-specific survival may be the errors resulting from the misclassification of the cause of death, while the main limitation of relative survival may be the unavailability of adequate life tables, especially in cancers in which regular screening is performed or in cancers with risk factors also associated with other causes of death, such as tobacco.

In studies comparing relative survival and cancer-specific survival, most cancers show a higher cancer-specific survival than relative survival, except for cancers detectable by screening. Prostate cancer shows the greatest difference in all these types of studies [24,25,26]. In the most recent study on this topic, which included more than 700,000 prostate cancer cases from 18 Surveillance, Epidemiology, and End Results (SEER) Program registries, the relative survival was 4.8% higher than the cancer-specific survival. In a study by Makkar et al. [25], the difference was 5.7%. However, the difference between relative survival and cancer-specific survival in prostate cancer in Mallorca was only of 2%, and this could indicate the high quality of our data on cause of death due to a meticulous review carried out of the medical histories.

Risk and treatment were the main factors associated to survival, but the histology and Charlson index were also independent prognostic factors. Age was not associated with survival after adjusting by treatment.

We chose risk instead of stage because the percentage of missing values in stage was too high (>50%). We could have assigned N or M values to complete stage [27], but the low percentage of missing values in risk made possible the use of MI, which many authors recommend and use in studying prostate cancer [27] and other types of cancer [16,28,29,30,31]. At the same time, the percentage of missing values in PSA was also very high, but we retrieved information about the risk in the clinical records, which takes into account the PSA value; therefore, it would have been redundant to include PSA in competing-risks regression.

We observed that risk was a strong predictor of survival. While survival in low-risk cases was 100%, very-high-risk cases presented a survival that was lower than that of the medium-risk cases by a factor of 16.72. The identification of these high-risk cases, which represent 23% of prostate cancer cases in Mallorca, more than in other series [32], is crucial, especially because treatment is also a strong predictor of survival. Therefore, high-risk prostate cancer cases warrant an early identification, and should be treated with an integrated approach including surgery, radiotherapy and systemic therapy [33].

Surprisingly, the choice of treatment depended mostly on age, not on risk or even on comorbidity. A similar result was observed by some authors [34], but others showed that this association between treatment and age could change in subsequent periods [12]. When we included the treatment variable, age fell out of the model and then the model fit better. Thus, our results could question the independent effect of age on prostate cancer survival obtained in other studies [11].

Comorbidity increases the risk of dying from other causes in prostate cancer, but, as we have shown in our study, the effects of comorbidity on prostate cancer CSS are not clear because survival worsened in cases with Charlson index of 2 but not in cases with Charlson index of 3 or above. The independent effect of comorbidity was found in other studies, some of them population-based [11,35]. The largest population-based study restrained to a specific stage [11] in the United States showed that a higher comorbidity score is generally associated with higher overall mortality and lower prostate cancer-specific mortality. In our case, the association with comorbidity is not clear. We hypothesize that the Charlson index is not a good marker of comorbidity in prostate cancer.

Based on material from the European Association of Urology [8], during the period of time in which the patients in this study were treated (from 2006 to 2011), the treatment recommendations were based on the stage and secondarily on the PSA, which translated to risk in the current guidelines. However, in our study we observed that treatment was more related to age and life expectancy, and therefore we believe that the follow-up of the recommendations would not have been desirable, as has already been seen in other studies [36] and in a previous study of ours [37].

The strength of this study is that the sample was population-based, and it included all categories of risk. We were able to follow some of the cases for up to nine years. Moreover, we knew the cause of death, which allowed us to calculate cause-specific survival. The application of the competing-risks model instead of the Cox model used in other studies [16] made it possible to obtain more adequate estimates of the risk of dying from prostate cancer, taking into account that there are patients who die from other causes. On the other hand, the use of multiple imputation made it possible to use all the patients in the database and obtain unbiased and more accurate estimates of prostate cancer survival.

The limitations of this study are related to the fact that the information came only from one cancer registry, so the external validity could be questioned. In addition, it would be desirable to have more recent data.

In summary, risk and treatment, and not age, were the main variables associated with survival of prostate cancer. The association between comorbidity and survival of prostate cancer is not clear.

## 5. Conclusions

In Mallorca, prostate cancer patients have very good 5-year survival. Nevertheless, in cases with a very high risk, which represent almost one in four prostate cancers, it drops from 91% to 69%. As such, their identification should be a priority.

Treatment is an independent prognostic factor of prostate cancer in Mallorca, and it is associated with age and life expectancy, but not with risk, as clinical guidelines recommend. Instead, age and comorbidity are not clear prognostic factors of prostate cancer.

## Figures and Tables

**Table 1:** Clinical description of prostate cancer cases diagnosed in Mallorca between 2006–2011 (N = 2921).

Variable	Categories	Number	%	% Valid	After MI
Age	15–54	163	5.6	5.6	
	55–64	817	28.0	28.0	
	65–74	1221	41.8	41.8	
	75–84	598	20.5	20.5	
	85 or +	122	4.2	4.2	
Histology	Adenocarcinoma	2710	92.8	92.8	
	Unspecified	161	5.5	5.5	
	Other	50	1.7	1.7	
T_PT	1	599	20.5	34.5	
	2	756	25.9	43.5	
	3	355	12.2	20.4	
	4	27	0.9	1.6	
	Missing	1184	40.5		
N_PN	0	1214	41.6	95.2	
	1	61	2.1	4.8	
	Missing	1646	56.4		
M	0	1908	65.3	90.9	
	1	190	6.5	9.1	
	Missing	823	28.2		
Stage	I	454	15.5	33.7	
	II	438	15.0	32.5	
	III	216	7.4	16.0	
	IV	240	8.2	17.8	
	Missing	1573	53.9		
PSA	≤10	1025	35.1	59.4	
	11–20	306	10.5	17.7	
	>20	395	13.5	22.9	
	Missing	1195	40.9		
Gleason	4	34	1.2	1.5	
	5	134	4.6	5.8	
	6	937	32.1	40.5	
	7 (3 + 4)	615	21.1	26.6	
	7 (4 + 3)	273	9.3	11.8	
	8	166	5.7	7.2	
	9	143	4.9	6.2	
	10	13	0.4	0.6	
	Missing	606	20.7		
Risk	Low	189	6.5	8.6	9.5
	Medium	512	17.5	23.2	24.9
	High	971	33.2	44.0	42.7
	Very high	533	18.2	24.2	22.9
	Missing	716	24.5		
Charlson index	0	1745	59.7	59.7	
	1	694	23.8	23.8	
	2	295	10.1	10.1	
	≥3	187	6.4	6.4	
Treatment	Local	1478	50.6	50.6	
	Systemic	642	22.0	22.0	
	Mixed	231	7.9	7.9	
	Observation/unspecified	570	19.5	19.5	
Vital status	Alive	2244	76.8	76.8	
	Death from prostate cancer	324	11.1	11.1	
	Death from other causes	349	12.0	12.0	
	Missing	4	0.1		

**Table 2:** Relationship of treatment with other variables.

Statistical Indexes	Risk	Charlson Index	Age	Life Expectancy	Histology
Cramer’s V index (*p* < 0.001)	0.144	0.090	0.321	0.510	0.206
Pearson’s Chi-Square (*p* < 0.001)	137.75	71.63	902.44	759.99	248.48

**Table 3:** Cause-specific survival function by years of follow-up and risk through actuarial method, before and after multiple imputation (m = 10).

	Original Set n = 2202					Imputed Set n = 2917				
Year	Low Risk	Medium Risk	High Risk	Very High Risk	Total	Low Risk	Medium Risk	High Risk	Very High Risk	Total
1	1.00	0.99	0.99	0.86	0.96	1.00	0.99	0.98	0.86	0.96
2	1.00	0.98	0.97	0.77	0.93	1.00	0.98	0.96	0.79	0.93
3	1.00	0.97	0.94	0.72	0.91	1.00	0.97	0.94	0.74	0.91
4	1.00	0.97	0.93	0.70	0.90	1.00	0.97	0.93	0.72	0.90
5	1.00	0.95	0.92	0.67	0.89	1.00	0.96	0.93	0.69	0.89
6	1.00	0.95	0.90	0.65	0.88	1.00	0.96	0.91	0.67	0.88
7	1.00	0.94	0.88	0.65	0.87	1.00	0.95	0.89	0.67	0.87
8	1.00	0.92	0.87	0.65	0.86	1.00	0.93	0.88	0.67	0.86
9	1.00	0.87	0.87	0.62	0.84	1.00	0.91	0.88	0.64	0.84

**Table 4:** Five year relative survival by risk before and after multiple imputation.

Risk	Original Set n = 2202	Imputed Set n = 2917
Total	0.91	0.91
Low risk	1.00	1.00
Medium risk	0.99	0.98
High risk	0.94	0.94
Very high risk	0.68	0.70

**Table 5:** Competing-risks regression model of prostate cancer diagnosed in Mallorca between 2006 and 2011 before (Model 1) and after (Model 2) multiple imputation (m = 10).

	Model 1 (Original Data Set) n = 2013		Model 2 (Imputed Data Set) n = 2728	
Variables	Subhazard Ratio	CI 95%	Subhazard Ratio	CI 95%
Charlson index (ref. 0)				
Charlson = 1	0.80	0.59, 1.08	0.84	0.62, 1.13
Charlson = 2	1.83	1.26, 2.66	1.58	1.11, 2.26
Charlson >= 3	1.40	0.91, 2.13	0.94	0.57, 1.56
Risk (ref. medium)				
High risk	2.68	1.67, 4.31	2.90	1.78, 4.71
Very high risk	17.53	11.08, 27.72	16.72	10.31, 27.09
Histology (ref. adenoc.)				
Unspecified	3.40	2.50, 4.61	4.78	3.55, 6.42
Others	4.01	2.11, 7.59	2.42	1.12, 5.20
Treatment (ref. local)				
Systemic	37.49	20.54, 68.42	29.83	16.83, 52.88
Mixed	10.60	5.16, 21.75	10.51	5.40, 20.46
Observation/unspecified	53.18	28.23, 100.19	35.17	19.36, 63.87

## Data Availability

Data available on request due to privacy/ethical restrictions.
